# Process Optimization and Microstructure-Property Regulation of P20 Plastic Mold Steels

**DOI:** 10.3390/ma19112423

**Published:** 2026-06-05

**Authors:** Luliang Zhao, Zhenguo Hou, Chunqiao Xing, Min Yang, Jie Yan, Ziwen Li, Zan Yao

**Affiliations:** 1 School of Materials Science and Engineering, Shanghai University, Shanghai 200444, China; 18115694113@163.com (L.Z.); 13673319742@163.com (Z.H.); 18642515957@163.com (C.X.); 19732612778@163.com (J.Y.); ziwenli2025@163.com (Z.L.); 2 Composite Research Center, Shanghai University, Shanghai 200444, China; ym2008@shu.edu.cn; 3 State Key Laboratory of Advanced Refractories, Shanghai University, Shanghai 200444, China

**Keywords:** plastic mold steel, quenching and tempering treatment, mechanical property, polishing performance

## Abstract

This study systematically investigated the effects of air-cooled pre-hardening and oil-quenched quenching-and-tempering processes on the microstructure, mechanical properties, and polishing performance of P20 plastic mold steel. Increasing the austenitizing temperature from 820 °C to 940 °C resulted in a more uniform carbide distribution, a slight improvement in hardness, and enhanced polishing performance for both processes. However, grain coarsening at 940 °C reduced the impact toughness from 157.6 J to 111.7 J. After tempering at 650 °C, both processes yielded a tempered sorbite microstructure. However, in the air-cooled samples, the carbides were aligned along the bainite lath direction, whereas in the oil-quenched samples, they exhibited an equiaxed, non-directional distribution owing to the complete recovery of the matrix. Austenitizing at 940 °C followed by air cooling and tempering at 550 °C provides the optimal balance of hardness, toughness, and polishing performance. Mitigating elemental segregation and narrowing the segregation bands represent key strategies for further enhancing polishing performance.

## 1. Introduction

Plastic mold steel is a type of steel specifically designed for manufacturing plastic molding dies, requiring properties such as corrosion resistance, uniform hardness, and good polishing performance. It is widely used in plastic molds for producing automotive components, electronic device housings, optical lenses, and other products. In recent years, the rapid growth of the automotive industry, coupled with increasingly stringent energy conservation and emission standards, has accelerated the shift toward vehicle lightweighting. For electric vehicles in particular, reducing body mass is essential for extending driving range, thereby further driving the adoption of lightweight plastic components. Consequently, the demand for plastic mold steel has risen steadily [1,2,3]. Furthermore, automotive components frequently necessitate superior surface finish; consequently, plastic mold steel must exhibit excellent polishing performance. In applications such as lamp housings and instrument panels, the corresponding molding dies are often required to achieve a mirror-like surface quality. The polishing behavior of plastic mold steel is critically influenced by microstructural features and metallurgical defects, including segregation, non-metallic inclusions, and porosity.

Plastic mold steels can be classified into various types based on their properties and manufacturing processes, including quenched and tempered, pre-hardened, carburized, age-hardening, and corrosion-resistant grades. Among these, quenched and tempered plastic mold steels require the steel mill to cut hot-rolled or hot-forged billets, reheat them to achieve an austenitic structure, and then subject them to quenching and tempering. In contrast, pre-hardened plastic mold steel involves the steel mill allowing hot-rolled or hot-forged billets to cool naturally to form a bainitic microstructure, followed by tempering at a specific temperature to achieve the hardness required for mold machining. Compared with quenched and tempered plastic mold steel, it is an energy-saving and economical product with wider application, mainly including grades such as AISI P20, AISI 718, DIN 1.2311, DIN 1.2738, and GB 3Cr2Mo [4,5,6]. For quenched and tempered as well as pre-hardened plastic mold steels, current research efforts mainly focus on optimizing their microstructure, mechanical properties, and polishing performance through appropriate alloying strategies and heat treatment processes optimization [7,8,9,10,11,12,13,14,15,16,17,18].

Liu et al. [7] demonstrated that adding 0.1 wt.% of V can enhance the strength, toughness and hardenability of 718H plastic mold steel through mechanisms such as precipitation strengthening and dislocation strengthening. However, excessive V may have an adverse effect on impact toughness. Hoseiny et al. [11] investigated the effects of different heat treatments on the mechanical and cutting properties of pre-hardened plastic mold steel and found that quenching and tempering treatment had a better effect. Wu et al. [15] investigated the influence of Ti addition on the continuous cooling transformation behavior, hardness uniformity, and microstructure of a prehardened plastic mold steel. Their results demonstrated that Ti effectively refined the grain size through grain boundary pinning and significantly retarded the nucleation of proeutectoid ferrite within the intercritical region. Long et al. [16] examined the martensitic microstructure of 20CrNi2Mo steel subjected to varying quenching temperatures and elucidated the correlation between microstructural characteristics and mechanical properties using the Hall–Petch relationship. Liu et al. [8] investigated the influence of tempering temperature on 718H plastic mold steel and observed that increasing the tempering temperature led to a progressive enhancement in fatigue resistance, accompanied by a concomitant reduction in yield strength, tensile strength, and hardness. Currently, the technical approaches to improving the surface roughness of plastic mold steels are mainly divided into two categories. First is optimizing polishing performance by controlling the microstructure through heat treatment. For example, Chi [2] investigated the relationship between the microstructure and polishing characteristics of large-section 1.2738 plastic mold steel. The study revealed that polishing performance was correlated with microstructure and composition. The polishing performance was found to be strongly dependent on the microstructure and composition, with tempered sorbite exhibiting the most favorable polishing behavior due to its fine and uniform morphology. In contrast, the presence of coarse carbide particles in tempered martensite and pearlite resulted in inferior polishing performance. Second, enhancing surface quality from the processing perspective using novel polishing techniques such as laser polishing and ultrasonic polishing. For instance, Zhang et al. [17] employed a hybrid polishing technique combining continuous and pulsed laser polishing, which significantly improved the material’s polishing performance. Shiou [18] employed ultra-precision surface finishing on NAK80 mold steel, achieving a surface roughness as low as 0.016 μm and thereby significantly enhancing its polishing quality.

For pre-hardened plastic mold steels, the rolling temperature and tempering temperature exert a significant influence on the characteristics of bainite and the subsequent tempered microstructure, as well as on the resulting mechanical performance. However, systematic investigations into these effects remain relatively limited. Meanwhile, no analysis has yet been reported on the relationship between alloying element segregation and polishing performance. To further elaborate on the microstructural and property differences between pre-hardening processes and tempering heat treatment, as well as the impact of solidification element segregation on polishing performance, in this work, P20 plastic mold steel was selected as the experimental material to simulate the prehardening process. The evolution of microstructure, hardness, and polishing performance was systematically investigated following austenitizing at 820–940 °C, subsequent air cooling, and tempering within the temperature range of 450–650 °C. These results are compared with those of specimens subjected to oil quenching followed by tempering, aiming to provide a theoretical basis for optimizing the pre-hardening process of plastic mold steel.

## 2. Materials and Methods

**Figure 1:**
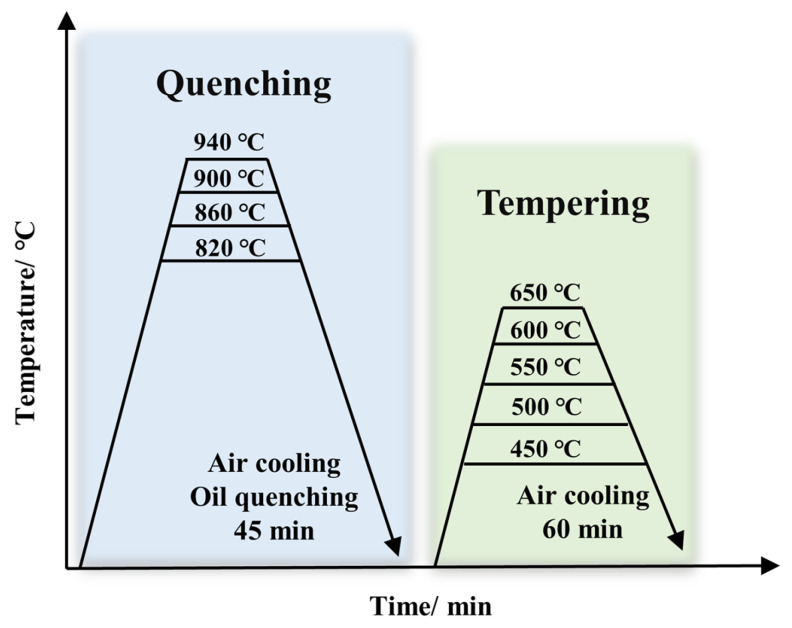
Heat treatment process diagram.

The P20 plastic mold steel used in this experiment was provided by a steel mill, and its standard chemical composition is shown in Table 1. Thermodynamic calculations were performed using JMatPro software (Version 13.0). The calculated critical temperatures are: A_c1_ is 732 °C, A_c3_ is 791 °C, the solidus temperature is 1445 °C, and the precipitation temperature for M_23_C_6_ carbides is 783 °C. Cubic specimens measuring 19 mm × 19 mm × 20 mm were cut and placed in a KSL-1500X-S box-type resistance furnace (Hefei Kejing Materials Technology Co., Ltd., Hefei, China) for heat treatment. The specific heat treatment process (Figure 1) is as follows: holding at 820 °C, 860 °C, 900 °C, and 940 °C for 45 min, then air cooling after the furnace to simulate the pre-hardening process and tempering at 550 °C. Under the same heating conditions of 820–940 °C, the samples were oil quenched and tempered at 550 °C, and the microstructure and properties were compared. The samples cooled in air or quenched in oil at 860 °C were selected for tempering at 450, 500, 550, 600, and 650 °C, and the differences in microstructure and properties were compared.

Following grinding and polishing, the heat-treated specimens were etched using a 4% nitric acid alcohol. The microstructural characteristics and elemental segregation resulting from the various heat treatment procedures were examined by means of a BH200M optical microscope (OM) (Ningbo Sunny Instruments Co., Ltd., Ningbo, China), an FEI Nova Nano SEM 450 scanning electron microscope (SEM) equipped with an energy-dispersive spectrometer (EDS) (FEI, Hillsboro, OR, USA), and a Talos F200X G2 transmission electron microscope (TEM) (FEI, Hillsboro, OR, USA). TEM sample preparation was conducted as follows: the specimen was first mechanically polished using silicon carbide abrasive paper down to a final thickness of approximately 50 μm. A 3 mm-diameter disc was then punched from the thinned region. Subsequently, electropolishing was performed via double-jet electropolishing in a solution consisting of 5 vol.% perchloric acid in ethanol, at a constant voltage of 25 V and a bath temperature of −20 °C. Phase identification of the carbides present within the specimens was performed using an X-ray diffractometer (XRD) (Bruker AXS GmbH, Karlsruhe, Germany) with Cu-Kα radiation (λ = 1.5406 Å). The diffraction patterns were recorded over a 2θ angular range of 10–90° at a scanning rate of 2°/min.

The hardness was measured by an HV-1000MPTA micro-Vickers hardness tester with a loading force of 1 kg and a loading time of 10 s (Shanghai Hengyi Precision Instrument Co., Ltd., Shanghai, China). Room-temperature impact toughness was evaluated for specimens subjected to different heating temperatures using a pendulum-type impact testing machine. Following the heat treatment procedures, all specimens were prepared under identical grinding and polishing conditions utilizing a YMPZ-1A-300 automatic grinding–polishing machine (Shanghai Yuguan Machinery Equipment Co., Ltd., Shanghai, China). The surface roughness of the prepared specimens was subsequently measured with an SJ-210 surface roughness tester (Mitutoyo Corp., Kawasaki, Japan). Three-dimensional surface topography of the sample polished surface was reconstructed employing a Bruker Contour GT-K 3D optical profilometer (Bruker Corp., Billerica, MA, USA).

To analyze the growth behavior of carbides during tempering, the classical Ostwald ripening formula [19] was employed:

(1) $$r^{3}-{r_{0}}^{3}=\left(\frac{8\gamma DCf^{2}}{9RT}\right)t^{3}$$

Among them, r represents the radius of precipitated carbides, $r_{0}$ is the initial radius of carbides, γ is the surface energy of carbides, $J/m^{2}$; D is the diffusion coefficient, $m^{2}/s$; f is the molar volume of carbides, $m^{3}/\mathrm{mol}$; C is the atomic fraction of carbides in the equilibrium state, $\mathrm{mol}/m^{3}$; T is the thermodynamic temperature, K; and t is the time, s.

## 3. Results and Discussion

### 3.1. Microscopic Structure Characterization

#### 3.1.1. Changes in Grain Size Under Different Heating Temperatures

**Figure 2:**
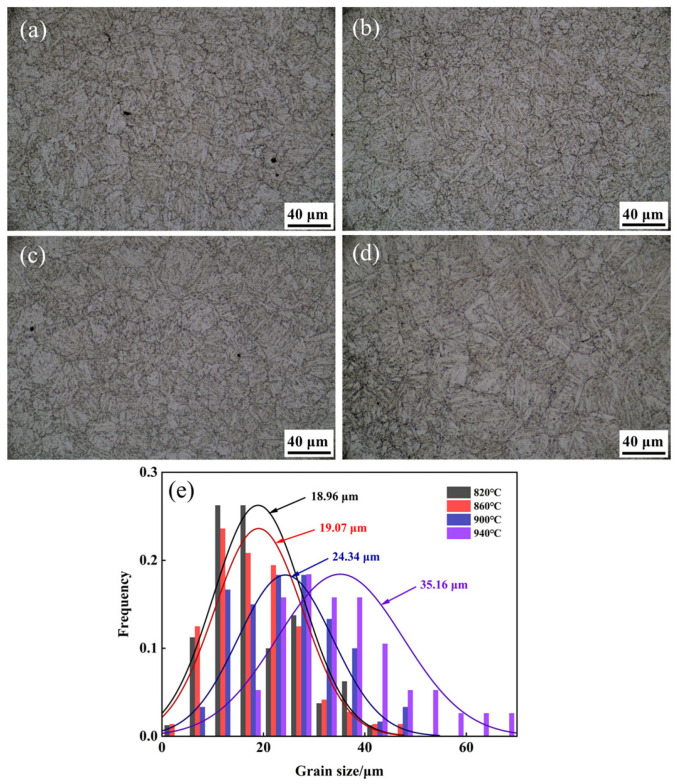
Microstructure and austenite grain size distribution of samples after austenitizing at different temperatures (820–940 °C), air cooling, and tempering at 550 °C (**a**) 820 °C; (**b**) 860 °C; (**c**) 900°C; (**d**) 940 °C; (**e**) grain size distribution curve.

Figure 2 shows the microstructure and grain size distribution of the samples tempered at 550 °C after being air-cooled at different temperatures ranging from 820 °C to 940 °C. The results indicate that as the heating temperature increases, the austenite grain size of the samples gradually increases. After heating at 820 °C, 860 °C, 900 °C, and 940 °C, the average austenite grain sizes of the samples are 18.96 μm, 19.07 μm, 24.34 μm and 35.16 μm respectively. When the austenitizing temperature is below 900 °C, the grain growth trend is relatively minor; however, when the temperature rises to 940 °C, the austenite grain size exhibited a more significant increase.

#### 3.1.2. Effects of Different Austenitizing Temperatures on Microstructure

**Figure 3:**
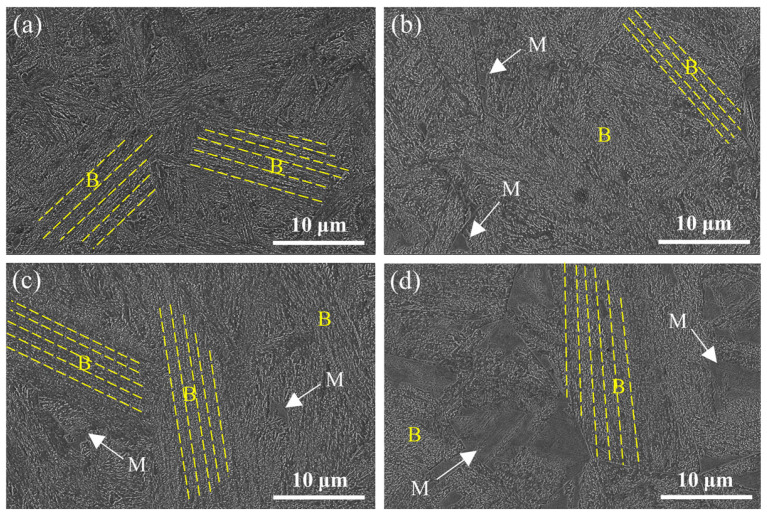
Microstructure under air cooled at different austenitizing temperatures (The dashed line represents the platelet morphology, B represents bainite, and M represents martensite): (**a**) 820 °C; (**b**) 860 °C; (**c**) 900 °C; (**d**) 940 °C.

Figure 3 presents the air-cooled microstructures of P20 plastic mold steel samples austenitized at 820–940 °C without tempering. It can be seen that the microstructure changes significantly with the increase in heating temperature. When the heating temperature is 820 °C, the microstructure of the sample is composed of fine lamellar bainite. As the temperature rises to 860 °C, the bainitic lamellar bundles coarsen, and the dissolution and homogenization of alloying elements enhance the stability of the austenite, leading to the appearance of a small fraction of blocky martensite and lower bainite in the air-cooled microstructure. When the temperature is further increased to 900 °C, both the lamellar bainite and martensite packets undergo additional coarsening. The lamellar bainite observed in the sample heated at 940 °C exhibits dimensions comparable to those at 900 °C; however, large blocky martensite islands appears in the microstructure, as indicated by the white arrows in Figure 3d.

**Figure 4:**
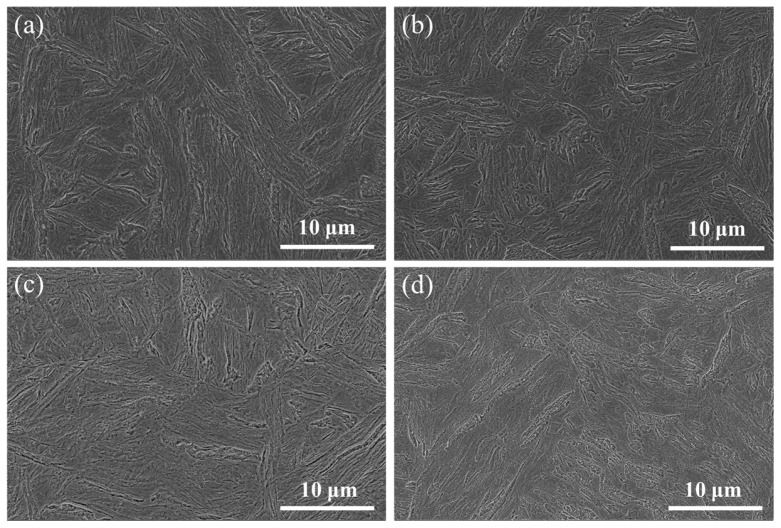
Microstructure after oil quenching at different austenitizing temperatures: (**a**) 820 °C; (**b**) 860 °C; (**c**) 900 °C; (**d**) 940 °C.

The microstructure of the P20 plastic mold steel samples at different oil-quenched temperatures ranging from 820 to 940 °C is shown in Figure 4. The main microstructure is lath martensite. As the quenching heating temperature increases, the laths of martensite gradually become longer and wider.

**Figure 5:**
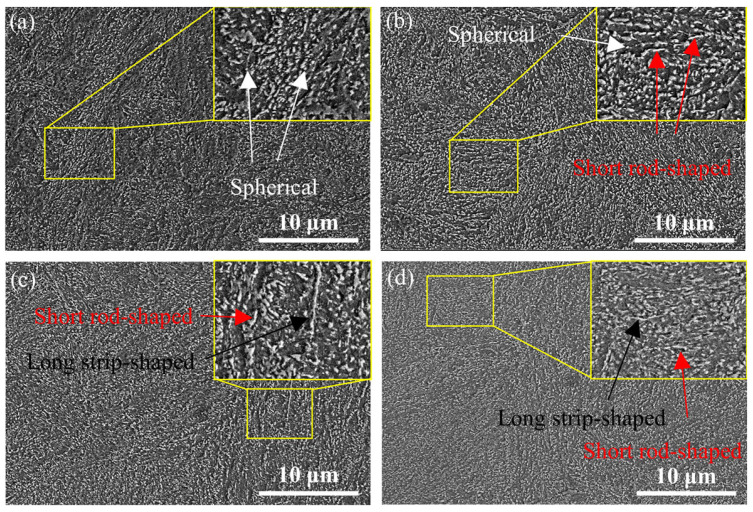
Microstructure of tempered specimens under air cooled at different austenitizing temperatures: (**a**) 820 °C; (**b**) 860 °C; (**c**) 900 °C; (**d**) 940 °C.

Figure 5 compares the microstructures of P20 steel samples air-cooled from 820 to 940 °C and subsequently tempered at 550 °C. The dominant microstructure is tempered sorbite, comprising ferrite laths embedded with spheroidized carbides. During the tempering process at a relatively high temperature of 550 °C, the original carbides in the air-cooled lamellar bainite and the fine carbides in the lower bainite will gradually spheroidize, forming spherical and short rod-shaped carbides. The presence of a small amount of long strip-shaped carbides in the microstructure is due to the precipitation of carbon elements in the supersaturated ferrite along the bainite dislocation lines.

**Figure 6:**
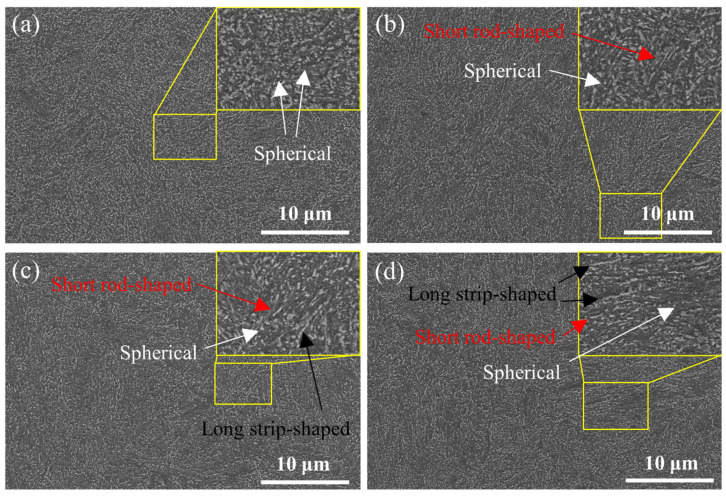
Tempering microstructure after oil quenching at different austenitizing temperatures: (**a**) 820 °C; (**b**) 860 °C; (**c**) 900 °C; (**d**) 940 °C.

Figure 6 shows the microstructure of the samples after oil quenching at 820–940 °C and tempering at 550 °C. The microstructure of the P20 oil quenched and tempered samples is also tempered sorbite, with a large number of spherical carbides dispersed (indicated by the white arrows in Figure 6). However, unlike the air-cooled tempered samples, these carbides nucleate and grow from the original quenched martensite matrix. As the carbides precipitate diffusely, the plate-like morphology of the quenched martensite weakens. With the increase in quenching heating temperature, short rod-shaped carbides appear in the matrix structure (indicated by the red arrows in Figure 6). When the quenching temperature exceeds 900 °C, some carbides will precipitate and grow along the boundaries of the martensite plates, and their morphology changes from short rods to long strips (indicated by the black arrows in Figure 6).

**Figure 7:**
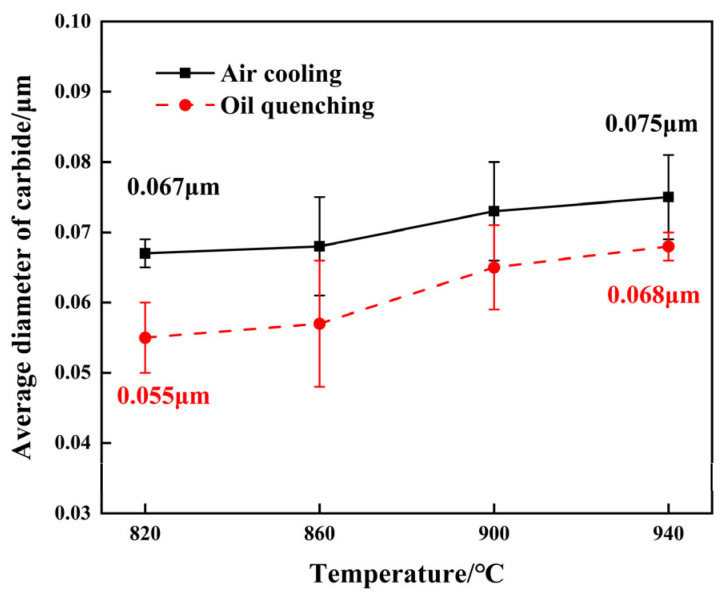
Equivalent diameters of carbides in samples that air-cooled and oil-quenched at different austenitizing temperatures, followed by tempered at 550 °C.

Figure 7 presents the statistical graph of the equivalent diameters of carbides in samples that have undergone air cooling and oil quenching at different austenitizing temperatures, followed by tempering at 550 °C. For each heat treatment condition, at least three SEM images were taken from randomly selected areas at a magnification of 30,000×. The equivalent diameter of carbides was measured using ImageJ software (Version 1.54g). It can be seen from the figure that with the increase in heating temperature, the equivalent diameters of carbides obtained by both processes show an increasing trend. At the same heating temperature, the carbide size of the air-cooled sample is about 20% larger than that of the oil-quenched sample. For both air-cooled and quenched samples, the increase in austenitizing temperature will make the diffusion of carbon and alloying elements such as Cr and Mo in the material more complete, and also increase the grain size of the alloy. These factors will reduce the nucleation sites and the nucleation rate of carbides during subsequent air cooling or tempering, and increase the size of carbides [9,20,21]. During the air cooling process after austenite heating, the cooling rate of the sample is relatively slow, which is conducive to the growth of carbides. Therefore, under the same heating temperature conditions, the carbide size in the air-cooled tempered sample is higher than that of the carbides nucleated and grown from the quenched martensite.

**Figure 8:**
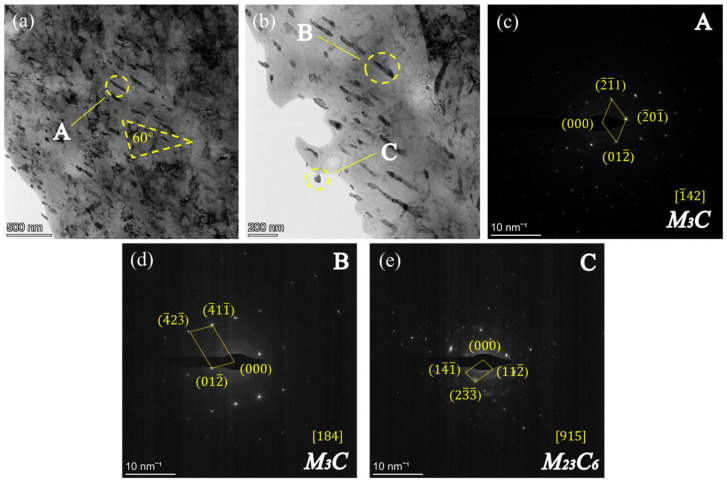
TEM images of specimens austenitized at 860 °C, air-cooled and tempered at 550 °C: (**a**,**b**) bright-field TEM images; (**c**,**d**,**e**) SAED patterns.

**Figure 9:**
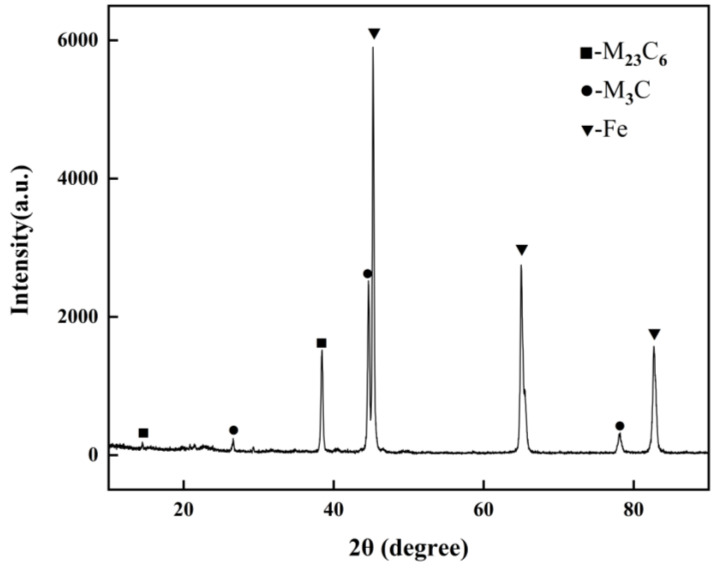
XRD patterns of specimens austenitized at 860 °C, air-cooled and tempered at 550 °C.

The crystal structure of the precipitated carbides in the samples air-cooled from 860 °C and tempered at 550 °C was characterized by TEM bright field images and selected area electron diffraction (SAED) patterns, as shown in Figure 8. The short rod-shaped carbides in Figure 8a,b were identified as M_3_C carbides, and the irregular spherical carbides in Figure 8b were M_23_C_6_ carbides. Additionally, the TEM bright field image in Figure 8a shows that the ferrite laths and the short rod-shaped carbides within the laths are inclined at 60°, which is a typical feature of tempered lower bainite [22]. Figure 9 presents the XRD pattern of the sample quenched at 860 °C and tempered, which is consistent with the TEM analysis results. Liu et al. [8] reported that Cr–Mo plastic mold steels near 550 °C tempering leads to the precipitation of M_3_C, M_23_C_6_, and Mo_2_C carbides. In the present study, however, Mo_2_C carbides were not detected. This is because, although Mo begins to diffuse appreciably in α-Fe at approximately 500 °C, Mo_2_C is a metastable phase that readily transforms during high-temperature tempering, rendering it difficult to detect. Furthermore, in the low-chromium (1% Cr) steel, M_7_C_3_ carbides possess higher thermal stability than M_23_C_6_, and consequently they were not observed either, which is consistent with previous work.

**Figure 10:**
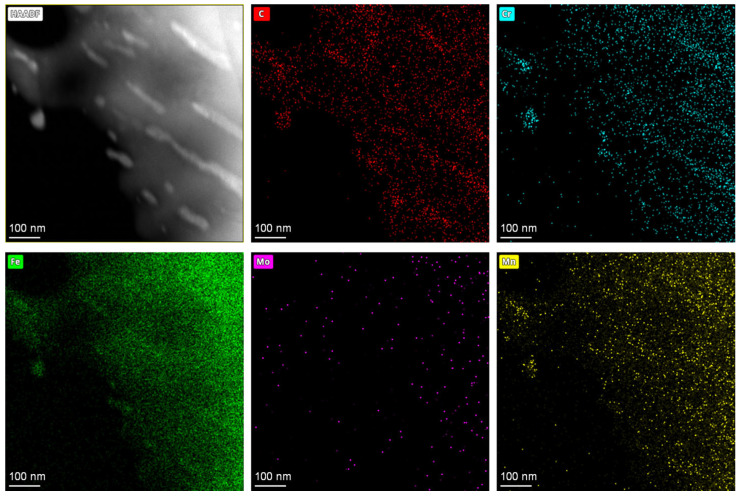
Surface scanning results for specimens austenitized at 860 °C, air-cooled and tempered at 550 °C.

Figure 10 shows the surface scanning results of the alloy element distribution of Figure 8b. The detection results indicate that both M_23_C_6_ and M_3_C carbides in the steel are Cr-rich carbides. Literature studies have shown [23] that the precipitation of Cr-rich M_23_C_6_ carbides in plastic mold steel will lead to a Cr-poor zone of about 10 nm around the carbides, but no similar phenomenon was found in this study [24,25].

#### 3.1.3. The Influence of Different Tempering Temperatures on the Microstructure

**Figure 11:**
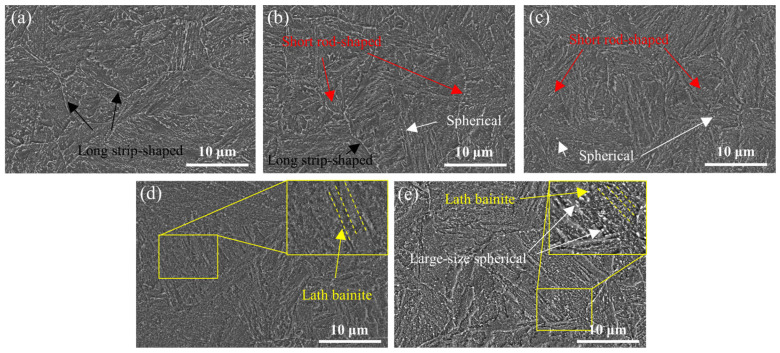
Microstructure after austenitized at 860 °C, air-cooled and tempered at different temperatures: (**a**) 450 °C; (**b**) 500 °C; (**c**) 550 °C; (**d**) 600 °C; (**e**) 650 °C.

Figure 11 shows the microstructure of samples after austenitized at 860 °C, air-cooled and subsequent tempering at various temperatures. All samples exhibit a tempered sorbite structure, characterized by numerous spherical carbides dispersed in the matrix. After tempering in the range of 450–650 °C, all samples still maintain the original morphology of bainite lamellar structure formed during air cooling. Even after tempering at 650 °C, carbides remain arranged parallel along the original bainite lamellar direction. In the samples tempered at 450–500 °C, a certain amount of long strip-shaped carbides are observed. These carbides originate from two mechanisms: retention of precipitates formed along prior austenite grain boundaries or bainitic interfaces during air cooling, and dissolution–precipitation of carbon atoms from supersaturated ferrite during tempering treatment. In contrast, specimens tempered at 550, 600, and 650 °C exhibit predominantly spherical and short rod-shaped carbides, with minimal occurrence of long strip-shaped variants. Moreover, with the increase in tempering temperature, the size of spherical carbides in the samples increases and coarsens, as indicated by the large carbide particles in Figure 11e.

**Figure 12:**
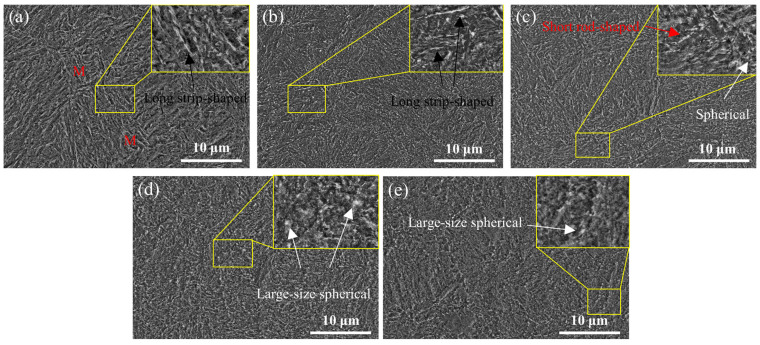
Microstructure after austenitized at 860 °C, oil-quenched and tempered at different temperatures: (**a**) 450 °C; (**b**) 500 °C; (**c**) 550 °C; (**d**) 600 °C; (**e**) 650 °C.

The microstructure of the samples after austenitized at 860 °C, oil-quenched and then tempered at 450–650 °C is shown in Figure 12. It is similar to that in the air-cooled and tempered samples. When tempered at lower temperatures ranging from 450 to 500 °C, a large number of thin lamellar carbides will nucleate and precipitate along the interface of the original quenched martensite plates, as shown in Figure 12a,b. This enables the microstructure of the sample to retain a relatively clear original quenched martensite plate morphology. As the tempering temperature increases, the carbon and alloy elements have a higher diffusion activation energy, and the carbon element desolidification rate accelerates. Spherical and short rod-shaped carbides precipitate both within the grains and at the plate interface, as shown in Figure 12c,d. The lath martensite undergoes significant recovery, and only a very small amount of carbonized particles with certain directionalities can be seen at the grain boundaries and plate interfaces. As the tempering temperature further increases to 650 °C, as shown in Figure 12e, the lath martensite is completely recovered, and the long strip-shaped carbides disappear. The microstructure transforms into tempered sorbite, similar to that of the air-cooled tempered samples. Some large-sized spherical carbide particles appear in the matrix.

According to Ostwald’s formula, the tempering temperature has a significant influence on the size of precipitated carbides [19], the formula of D and C will increase exponentially with the increase in tempering temperature. Therefore, as the tempering temperature rises, the size of carbides significantly increases, as shown in Figure 11.

### 3.2. Mechanical Property

**Figure 13:**
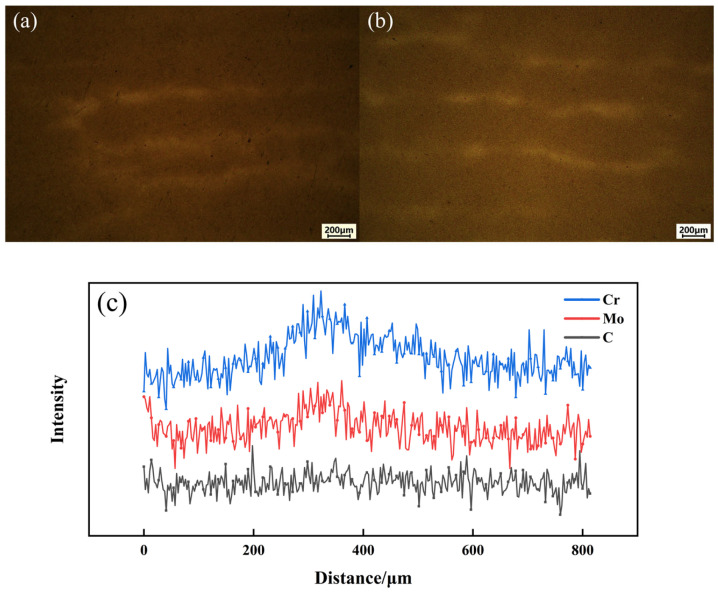
(**a**) Microstructure of the air-cooled sample at 860 °C and tempered at 550 °C; (**b**) microstructure of the oil-quenched sample at 860 °C and tempered at 550 °C; (**c**) element segregation line scanning in the segregation bands of the air-cooled sample at 860 °C and tempered at 550 °C.

**Figure 14:**
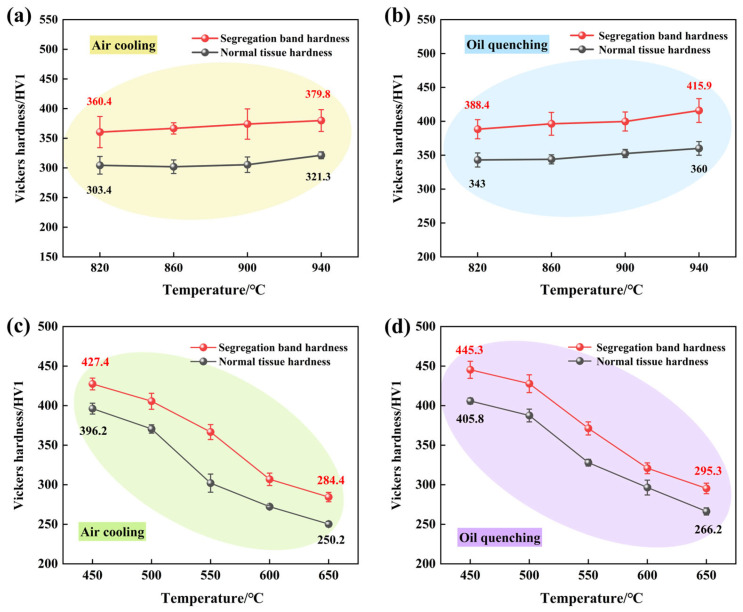
The Vickers hardness of the segregation bands and the matrix microstructure of the specimens under different heat treatment processes: (**a**) Air-cooled at 820–940 °C, followed by tempering at 550 °C; (**b**) oil-quenched at 820–940 °C, followed by tempering at 550 °C; (**c**) air-cooled at 860 °C, followed by tempering at 450–650 °C; (**d**) oil-quenched at 860 °C, followed by tempering at 450–650 °C.

Composition segregation is an inevitable phenomenon during the solidification process of alloy steel. P20 die steel is also prone to composition segregation during continuous casting after smelting. After rolling and cooling, segregation band-like structures will form. The typical segregation morphology of P20 is shown in Figure 13a,b. Combined with the line scan results in Figure 13c, it can be known that the white band-like structure is mainly caused by the segregation of Cr and Mo elements. Figure 14 shows the comparison of Vickers hardness of samples after different heat treatment processes. Each sample was measured 10 times and the average value was taken. Due to the segregation of alloying elements in the banded structure, its hardness is different from that of the matrix structure, so they were tested separately. As shown in Figure 14a,b, the Vickers hardness of samples heated to temperatures ranging from 820 to 940 °C, followed by either air cooling or oil quenching and subsequent tempering at 550 °C, increases with the austenitizing temperature. For the air-cooled samples, the hardness of the segregation bands increases from 360.4 HV1 to 379.8 HV1, and the hardness of the matrix increases from 303.4 HV1 to 321.3 HV1. For the oil-quenched samples, the hardness of the segregation bands increases from 388.4 HV1 to 415.9 HV1, and the hardness of the matrix increases from 343 HV1 to 360 HV1. It is also noted that the overall hardness of the air-cooled samples is lower than that of the oil-quenched samples. Temperature is the primary driving force for atomic diffusion during heating. The higher the temperature, the faster and farther carbon and alloying elements such as chromium and molybdenum diffuse in austenite. Therefore, as the heating temperature increases, the elemental distribution within the austenite grains becomes more homogeneous, which in turn promotes a more uniform precipitation of carbides during tempering, as illustrated in Figure 5 and Figure 6. The carbide distribution in the sample heated at 940 °C is more uniform and dispersed than that in the sample heated at 820 °C, and the macroscopic hardness of the material increases. However, it is not the case that the higher the heating temperature, the better. When the heating temperature exceeds a certain threshold, the grains will grow and coarsen, and the total area of grain boundaries will decrease, resulting in a decline in the toughness and strength of the steel [26,27,28].

In contrast, as shown in Figure 14c,d, the hardness of samples tempered at temperatures ranging from 450 to 650 °C after either air cooling or oil quenching from 860 °C decreases in a nearly linear manner. For the air-cooled samples, the segregation bands decreased from 427.4 HV1 to 284.4 HV1 and the matrix decreased from 396.2 HV1 to 250.2 HV1; for the oil-quenched samples, the segregation bands decreased from 445.3 HV1 to 295.3 HV1 and the matrix decreased from 405.8 HV1 to 266.2 HV1. The hardness decline trends of the two processes during tempering are similar. As the tempering temperature increases, the carbides spheroidize and coarsen, while the increased thermal activation energy promotes the migration and annihilation of dislocations, thereby reducing the dislocation density. Consequently, the hardness of the samples decreases significantly [29,30].

**Figure 15:**
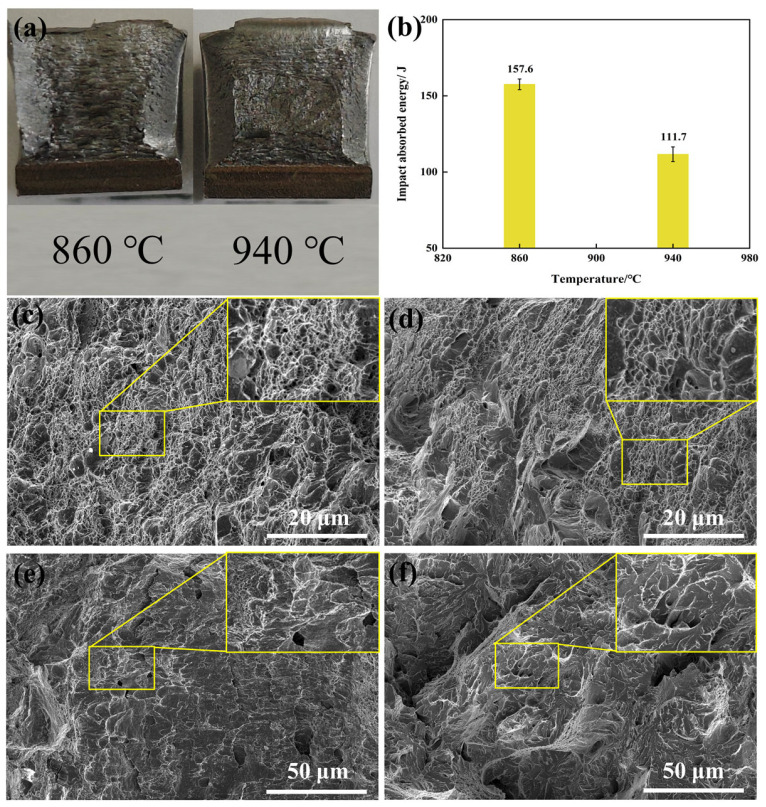
Impact energy and fracture morphology after different heat treatments: (**a**) Impact on the macroscopic fracture morphology; (**b**) impact energy of tempered specimens at different air-cooled temperatures; (**c**) fiber area of 860 °C air-cooled tempered specimens; (**d**) fiber area of 940 °C air-cooled tempered specimens; (**e**) radial area of 860 °C air-cooled tempered specimens; (**f**) radial area of 940 °C air-cooled tempered specimens.

As stated in the previous microstructure testing results, when the heating temperature increases, the growth trend of the sample grains in the temperature range of 820–900 °C is relatively small, but it will increase significantly at 940 °C. Therefore, samples subjected to air-cooled from 860 °C and tempered at 550 °C were compared with those air-cooled from 940 °C and tempered at the same temperature for evaluation of room-temperature impact toughness. Using V-shaped standard impact specimens, each process was tested three times to obtain the average value. Among them, the room-temperature impact energy of the 860 °C air-cooled sample reached 157.6 J, while the impact energy of the 940 °C sample decreased to 111.7 J. Figure 15a shows a comparison of the typical macroscopic morphology of the impact fracture surfaces from samples heated at two different temperatures. The fracture surfaces of the samples mainly consist of three parts: the fibrous area at the bottom of the notch, the rapidly expanding radial area, and the final shear lip area. The fibrous area and the shear lip area account for more than 80% of the total. Both test samples exhibit obvious ductile fracture characteristics, but a comparison also reveals that the radial area in the 940 °C air-cooled sample is higher than that in the 860 °C sample [31,32,33,34]. Field emission scanning electron microscopy was employed to examine the micro-morphology of the fibrous and radial areas on the fracture surfaces of impact specimens processed under the two conditions, as shown in Figure 15c–f. As shown in Figure 15c,d, the fibrous areas of crack initiation in both specimens contain numerous dimples, indicative of good material toughness. At the same time, through comparison, it was also found that the dimples in the 860 °C heated sample are smaller and deeper than those in the 940 °C sample. In addition, there are also a certain number of larger oval-shaped dimples in the fibrous area of the 940 °C heated sample, along with a small amount of cleavage steps and river-like cleavage pattern planes. During impact testing, the tearing and fracture of dimples in the crack initiation area require the absorption of substantial energy; consequently, the specimen heated at 860 °C exhibits superior impact toughness. Figure 15e,f show the morphology of the radial crack propagation area in the test specimens. Both test samples have typical cleavage river-like patterns on the fracture surfaces, and there are some small polygonal fracture surfaces at the river steps. Through comparison, it was found that the cleavage patterns in the 860 °C air-cooled sample are smaller, and the cracks formed more branches during the expansion process. Some cleavage fractures were replaced by tear, dimple, and other ductile fracture modes, and the material absorbed more energy; thus, its impact energy is higher than that of the 940 °C sample [35,36,37].

### 3.3. Polishing Performance

High-precision automatic grinding and polishing machines were used to perform grinding and polishing on different heat-treated samples. The specific mechanical grinding and polishing parameters are shown in Table 2.

**Figure 16:**
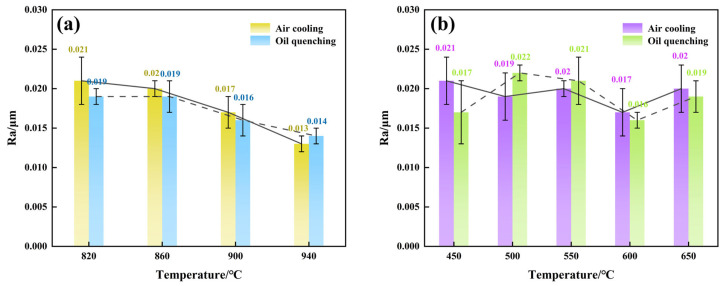
Surface roughness of samples under different heat treatment processes: (**a**) Air-cooled or oil-quenched at 820–940 °C, followed by tempering at 550 °C; (**b**) air-cooled or oil-quenched at 860 °C, followed by tempering at 450–650 °C.

Figure 16 shows the surface roughness (Ra) measured by the SJ-210 roughness tester under different heat treatments. For each heat treatment condition, the polished surface was measured at three different locations. The arithmetic mean roughness (Ra) was recorded for each location. As can be seen from Figure 16a, at the same heating temperature of 820–940 °C, the surface roughness between the air-cooled sample and the oil-quenched sample is similar. As the heating temperature increases, the surface roughness gradually decreases, and the material’s polishing performance improves. As discussed in the preceding microstructural analysis, this can be primarily attributed to the enhanced diffusion of alloying elements at elevated heating temperatures, which promotes the formation of a more homogeneous microstructure upon tempering and consequently improves the polishing performance of the material [38,39]. From Figure 16b, it can be seen that different tempering temperatures have little effect on the polishing performance of the air-cooled or oil-quenched P20 plastic mold steel. The surface roughness remains at approximately 0.02 μm.

**Figure 17:**
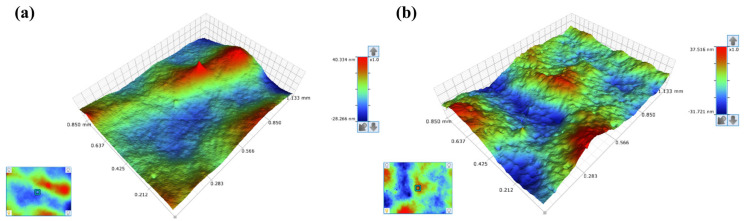
The three-dimensional surface morphology of the polished samples: (**a**) Air-cooled at 820 °C, followed by tempering at 550 °C; (**b**) air-cooled at 940 °C, followed by tempering at 550 °C.

Figure 17 presents the three-dimensional surface morphology of the polished samples subjected to air cooling from either 820 °C or 940 °C and subsequent tempering at 550°C. The detection results of the surface roughness value are basically consistent with those measured by the SJ-210 roughness tester. Meanwhile, it is found from Figure 17 that there are obvious fluctuations on the surface of the samples. The height difference between the highest point and the lowest point is approximately 70 nm, and the distance is about 200 μm. By comparing with the width of the segregation bands structure in Figure 13, it is found that they are basically of the same size. Therefore, it is reasonably speculated that the higher area (red area) in Figure 17a,b corresponds to the 3D morphology of the harder segregation bands structure in the material, and the lower area (blue area) is the matrix structure of the material.

Under identical polishing conditions, the hardness of a material is generally inversely proportional to its surface roughness. This is because the penetration depth of abrasive particles into the specimen surface decreases as the material hardness increases. A higher matrix hardness results in shallower abrasive penetration, producing finer and shallower scratches together with more uniform material removal, which ultimately yields a lower surface roughness. In the present experiment, as the austenitizing temperature increased, the tempered matrix hardness rose from approximately 303.4 HV1 (air-cooled from 820 °C) to about 321.3 HV1 (air-cooled from 940 °C), and the corresponding surface roughness Ra decreased from 0.021 μm to 0.013 μm, consistent with the trend described above. However, when the overall hardness distribution of the specimen is non-uniform, softer regions are preferentially abraded during polishing, whereas harder areas protrude slightly, ultimately causing surface defects such as “orange peel” and “pitting” on the polished mirror surface and severely degrading the polishing performance. Therefore, reducing element segregation during the solidification process, reducing the width of the band structure, and reducing the microscopic organization hardness difference caused by element segregation will be beneficial to improving the mirror polishing performance of the material [40,41]. Further research on the improvement of segregation in pre-hardened plastic mold steel will be carried out.

## 4. Conclusions

This paper investigates the effects of different heat treatment processes on the microstructure, carbide precipitation, mechanical properties and polishing performance of P20 plastic mold steel. The following conclusions are drawn:

(1) Both the air-cooled pre-hardening route and the oil-quenched quenching-and-tempering route yield a tempered sorbite microstructure after tempering at 550 °C. Raising the austenitizing temperature promotes the diffusion of alloying elements, resulting in a more uniform carbide distribution and simultaneous improvements in hardness and polishing performance. However, austenitizing at 940 °C causes grain coarsening, which leads to a slight decrease in impact toughness.
(2) As the tempering temperature rises from 450 °C to 650 °C, the carbides undergo spheroidization and coarsening, leading to a linear decrease in hardness. At 650 °C, the carbides in the air-cooled specimens become aligned along the bainitic lath direction, whereas those in the oil-quenched specimens exhibit an equiaxed, non-directional distribution owing to complete recovery of the matrix.
(3) Due to the segregation of Cr and Mo during solidification, P20 mold steel develops banded structures after rolling and cooling. Observing 3D morphology in conjunction with polishing mechanisms, reducing the width of banded structures and minimizing elemental segregation are key directions for further improving polishing performance in the future.
(4) Considering the overall properties of microstructure, hardness, impact toughness, and polishability, the optimal pre-hardening process parameters for P20 plastic mold steel are austenitizing at 940 °C followed by air cooling and tempering at 550 °C.

## Figures and Tables

**Table 1:** Standard chemical composition (wt.%) of P20 plastic mold steel.

C	Si	Mn	P	S	Cr	Mo	Ni
0.28–0.4	0.2–0.8	0.6–1.0	≤0.03	≤0.03	1.4–2.0	0.3–0.55	0.05–0.1

**Table 2:** Mechanical polishing parameters.

Step	Medium	Time/min	Grinding Disc Speed/rpm	Platen Pressure/N	Sample Rotation Speed/rpm
1	120 sandpaper	3	300	12	50
2	320 sandpaper	3	300	12	50
3	600 sandpaper	3	300	12	50
4	1000 sandpaper	3	300	12	50
5	1500 sandpaper	3	300	12	50
6	W 0.5 polishing paste	6	300	12	50

## Data Availability

The original contributions presented in this study are included in the article. Further inquiries can be directed to the corresponding author.
